# Potential inhibition of major human cytochrome P450 isoenzymes by selected tropical medicinal herbs—Implication for herb–drug interactions

**DOI:** 10.1002/fsn3.789

**Published:** 2018-11-19

**Authors:** Segun Johnson Showande, Titilayo Oyelola Fakeye, Marena Kajula, Juho Hokkanen, Ari Tolonen

**Affiliations:** ^1^ Faculty of Pharmacy Department of Clinical Pharmacy and Pharmacy Administration University of Ibadan Ibadan Nigeria; ^2^ Admescope Ltd Oulu Finland

**Keywords:** chronic diseases, cytochrome P450 isoenzymes, herb–drug interactions, in vitro assay, medicinal herbs, traditional medicines

## Abstract

**Background:**

Increasing use of medicinal herbs as nutritional supplements and traditional medicines for the treatment of diabetes, hypertension, hyperlipidemia, and malaria fever with conventional drugs poses possibilities of herb–drug interactions (HDIs). The potential of nine selected widely used tropical medicinal herbs in inhibiting human cytochrome P450 (CYP) isoenzymes was investigated.

**Materials and methods:**

In vitro inhibition of eight major CYP isoenzymes by aqueous extracts of *Allium sativum, Gongronema latifolium, Moringa oleifera*,* Musa sapientum, Mangifera indica, Tetracarpidium conophorum*,* Alstonia boonei, Bauhinia monandra,* and *Picralima nitida* was estimated in human liver microsomes by monitoring twelve probe metabolites of nine probe substrates with UPLC/MS‐MS using validated N‐in‐one assay method.

**Results:**

*Mangifera indica* moderately inhibited CYP2C8, CYP2B6, CYP2D6, CYP1A2, and CYP2C9 with IC_50_ values of 37.93, 57.83, 67.39, 54.83, and 107.48 μg/ml, respectively, and *Alstonia boonei* inhibited CYP2D6 (IC_50_ = 77.19 μg/ml). *Picralima nitida* inhibited CYP3A4 (IC_50_ = 45.58 μg/ml) and CYP2C19 (IC_50_ = 73.06 μg/ml) moderately but strongly inhibited CYP2D6 (IC_50_ = 1.19 μg/ml). Other aqueous extracts of *Gongronema latifolium, Bauhinia monandra,* and *Moringa oleifera* showed weak inhibitory activities against CYP1A2. *Musa sapientum, Allium sativum,* and *Tetracarpidium conophorum* did not inhibit the CYP isoenzymes investigated.

**Conclusion:**

Potential for clinically important CYP‐metabolism‐mediated HDIs is possible for *Alstonia boonei, Mangifera indica,* and *Picralima nitida* with drugs metabolized by CYP 2C8, 2B6, 2D6, 1A2, 2C9, 2C19, and 3A4. Inhibition of CYP2D6 by *Picralima nitida* is of particular concern and needs immediate in vivo investigations.

## 1 INTRODUCTION

Management of chronic diseases is burdensome to patients (Eton et al., 2013; Ørtenblad, Meillier, & Jønsson, 2017) who tend to seek alternative remedies to conventional medications that may supposedly provide cure or offer safe use (Yarney et al., 2013; Joeliantina, Agil, Qomaruddin, Jonosewojo, & Kusnanto, 2016) This self‐medication practice is common among chronically ill patients or patients with terminal diseases (Bodenheimer, Lorig, Holman, & Grumbach, 2002; Hasan, Ahmed, Bukhari, & Loon, 2009), and the major culprit in this group of patients are dietary supplements and herbal medicines (Gardiner, Graham, Legedza, Eisenberg, & Phillips, 2006; Gardiner, Phillips, & Shaughnessy, 2008). The perceived believe of cure and safety of these agents makes ambulatory patients take them without due recourse to their physician (Mehta, Gardiner, Phillips, & McCarthy, 2008; Zhang, Onakpoya, Posadzki, & Eddouks, 2015).

In various settings, about 40%–57% of patients fail to disclose herbs used concomitantly with their medications to the physician (Robinson & McGrail, 2004; Fakeye, Tijani, & Adebisi, 2008; Kennedy, Wang, & Wu, 2008). Some of these herbs especially in sub‐Saharan Africa, India, and in some other parts of the world are consumed as seasoning agents (garlic—*Allium sativum*), vegetables (*Gongronema latifolium* and *Moringa oleifera*), and as edible fruits and seeds (*Musa sapientum, Mangifera indica, and Tetracarpidium conophorum*). Patients who are on medications and consumes these medicinal herbs may not be aware of potential herb–drug interactions (HDIs) that may occur. Others such as *Alstonia boonei, Bauhinia monandra,* and *Picralima nitida* are frequently used in sub‐Saharan Africa and India in the management of chronic diseases such as hypertension, diabetes, asthma, peptic ulcer, and cancer, as antimalarials and antimicrobials and other minor ailments (Mahomoodally, 2013; Ezuruike & Prieto, 2014; Iwu, 2014).

Sometimes, in the treatment of these diseases, herbs may be used singly or in combination and prepared as dried powder or decoction. There are many examples of such practice. For example, for rheumatism, bark of *Picralima nitida*, leaves of *Allium ascalonicum*,* Calliandra portoricensis,* and *Xylopia aethiopica* are grounded, cooked, and 15 ml of the mixture is mixed with corn pap and taken once daily (Olorunnisola, Adetutu, & Afolayan, 2015). For anemia, a mixture of the bark of *Mangifera indica* and fruits of *Aframomum melequeta* is dried and powdered with one tablespoonful administered daily (Gbadamosi, Moody, & OYekini, 2012). A decoction of dried bark of *Alstonia boonei* is also taken two‐ to three times daily by diabetic patients to lower blood glucose (Adotey, Adukpo, Opoku Boahen, & Armah, 2012).

Many diseases are usually managed or treated with conventional medicines which are mostly metabolized by cytochrome P450 (CYP) isoenzymes (Rendic & Guengerich, 2014). These isoenzymes are responsible for the metabolism of over 70% of prescription and over‐the‐counter medications (Rendic & Guengerich, 2014). Concomitantly administered drug may modulate these CYP isoenzymes activities leading to clinically significant drug–drug interactions. Such interaction may result in serious adverse drug reactions requiring hospitalization, especially drugs with narrow therapeutic index such as carbamazepine, theophylline, digoxin warfarin, and phenytoin (Greenblatt & von Moltke, 2005; Perucca, 2006; de Leon, 2007; Patel, Rana, Suthar, Malhotra, & Patel, 2014). It is thus rational to expect herbs to also elicit similar HDIs via modulation of cytochrome P450 isoenzymes when concomitantly administered with conventional drugs.

Herbs contain bioactive secondary metabolites such as anthocyanins, flavonoid, tannins, saponins, alkaloids, and cardenolides, some of which have been shown to inhibit the activity of CYP isoenzymes (Henderson, Miranda, Stevens, Deinzer, & Buhler, 2000; Dreiseitel et al., 2008; Kimura, Ito, Ohnishi, & Hatano, 2010; Sand et al., 2010). Several studies have documented the occurrence of serious adverse events as a result of concurrent administration of herbs and drugs. Such interactions included bleeding experienced with coadministration of *Allium sativum* and nonsteroidal anti‐inflammatory drugs (Fugh‐Berman, 2000), manic episode with *Panax ginseng* and phenelzine (Fugh‐Berman, 2000), and fatal seizure with concomitant administration of *Ginkgo biloba* and valproate (Kupiec & Raj, 2005). Several herbs such as St. John's wort, grapefruit juice, *Ginkgo biloba*, black pepper, and Echinacea are known inhibitors of CYP 2C9, 2C19, 2E1, and 3A4 (Wang et al., 2001; Gorski et al., 2004; Mohutsky, Anderson, Miller, & Elmer, 2006; Sukkasem & Jarukamjorn, 2016). Little is known of the effect of most tropical medicinal herbs on the metabolic capacity of CYP isoenzymes.

The possibility of occurrence of HDIs when herbs are consumed as seasoning agents, vegetables, fruits and as herbal medicines with conventional drugs informed this study. Since inhibition of CYP isoenzymes is the first step toward elucidating potential HDIs, this study evaluated nine widely used tropical medicinal herbs with a view to identifying those with potentials for CYP‐metabolism‐mediated HDIs.

Some of the herbs considered in this study have also been shown to inhibit CYP isoenzymes. *Allium sativum* inhibits CYP 2C9, 2C19, 2D6, 3A4/5, and 3A7 (Foster et al., 2001; Greenblatt, Leigh‐Pemberton, & von Moltke, 2006; Engdal & Nilsen, 2009); *Moringa oleifera*—CYP 1A2, 3A4, and 2D6 (Monera, Wolfe, Maponga, Benet, & Guglielmo, 2008; Taesotikul, Navinpipatana, & Tassaneeyakul, 2010; Ahmmed et al., 2015; Awortwe, 2015); *Musa sapientum*—CYP 1A2 and 3A11 (Chatuphonprasert & Jarukamjorn, 2012) while *Mangifera indica* has been shown to inhibit CYP 1A2, 2C9, 2C19, 2D6, 3A4, and 3A11 (Chatuphonprasert & Jarukamjorn, 2012). In the above studies, different enzyme sources, probe substrates, and extraction methods were employed in evaluating one to five CYP isoenzyme inhibition profiles of the stated herbs which do not represent the full complement of major human CYP isoenzymes. The present study improved on this by mimicking local methods of preparation of plant extracts, used pooled human liver microsomes containing eight major CYP isoenzymes responsible for over 70% metabolism of drugs (Rendic & Guengerich, 2014), and nine probe substrates with N‐in‐one cocktail method to profile the inhibitory potentials of the herbs on CYP isoenzymes.

## 2 MATERIALS AND METHODS

### 2.1 Preparation of aqueous extracts of plant parts

Nine medicinal herbs widely used in the tropics for the management of chronic diseases such as hypertension, diabetes, dyslipidemia, chronic kidney disease were selected for this study with the plant parts used and listed in Table 1. Plant parts used were sourced from open market and herb vendors. Some were identified and authenticated at the Department of Pharmacognosy, University of Ibadan with voucher numbers DPHUI 1385, DPHUI 1386, DPHUI 1387, DPHUI 1388, and DPHUI 1624 for *Alstonia boonei, Mangifera indica, Musa sapientum, Bauhinia monandra*, and *Moringa oleifera* leaves, respectively. Other plants parts such as *Tetracarpidium conophorum seeds, Picralima nitida* seed, *Allium sativum* bulb, and *Gongronema latifolium* leaves were collected and identified at Forestery Research Institute of Nigeria with voucher numbers No. FHI110276, FHI210232, FHI210233, and FHI210234, respectively. Plant parts were chopped into smaller bits and dried in the oven (Gallenhamp oven, Cat number GVH 500.010 G. Germany) separately at 40°C for 24–48 hr until a constant weight was obtained. Thereafter, each plant part was blended with Kenwood blender (model number: OWBL436003, China) for 3 min.

**Table 1:** Detail of medicinal herbs used in the screening of potential inhibition of cytochrome P450 isoenzymes

Name of herb	Family name	Part used	Local name	Common name	Uses^a^	Usual human dosage of dried powdered material
*Alstonia boonei* De Wild	Apocynaceae	Stem bark	Ahun (Y) Egbu (I)	Stoolwood Devil tree	Antimalarial, Aphrodisiac, Antidiabetic, Antimicrobial, and Antipyretic	1.5–3 g^b^
*Bauhinia monandra* Kurz	Leguminosae	Leaves	Abefe (Y)	Pink orchid Napoleon's plume	Pesticidal, Antimicrobial, Laxative, Antidiabetic	3–6 g^c^
*Musa sapientum* Linn	Musaceae	Unripe fruits	Ogede (Y) Abrika (I), Ayaba (H)	Banana	Antidiabetic, Anemia, Hypertension, Ulcers	1–4 g^c^
*Tetracarpidium conophorum* (Mull.‐Arg.) Hutch. & Dalz.	Euphorbiaceae	Seeds	Asala (I)	Walnut	Antidiabetic, Anti‐ulcer, Antimicrobial, Anitihypercholesterolemia.	5–8g^d^
*Gongronema latifolium* Benth.	Asclepiadaceae	Leaves	Madunmaro (Y) Utazi (I) Arokeke (Y),	Amaranth globe.	Antidiabetic, Antimicrobial, Antiparasitic, Hypertension, Laxative, Analgesic, Fevers	2–4 g^e^
*Picralima nitida* (Stapf) T. Durand and H. Durand	Apocynaceae	Seeds	Mkpokiri or Otosu (I) Abere (Y)	Akuamma plant	Antidiabetic, Jaundice, Pneumonia, Malaria, Hypertension	3–4 g^e^
*Mangifera indica* Linn	Anacardiaceae	Stem bark	Mangoro (Y)	Mango	Antidiabetic, Malaria, Analgesic, Diarrhea, Menorrhagia, Hypertension, Anemia,	1–3 g^c^
*Moringa oleifera* Lam.	Moringaceae	Leaves	Ewe igbale (Y), Zogale (H), Okwe‐oyibo (I),	Drumstick or Horse radish tree, Moringa	Antidiabetic, Anti‐cancer, Anemia, Hypertension, Aphrodisiac.	1–3 g^c^
*Allium sativum* L.	Amaryllidaceae	Bulb	Aayu (Y), Ayo‐ishi (I), Tafarunua (H)	Garlic	Antidiabetic, Hypertension, Hemorrhoids, Tumors	0.4–1.2 g^f^ or 3–5 g^c^

I, Igbo; Y, Yoruba; H, Hausa.

^a^Ezuruike & Prieto, 2014; ^b^Pharmasearch, 2006; ^c^Khare, 2011; ^d^Nnorom, Enenwa, & Ewuzie, 2013; ^e^Neuwinger, 1996; ^f^Federal Ministry of Health and World Health Organization, 2008.

Three hundred gram each of dried and powdered *Alstonia boonei* stem bark*, Mangifera indica* stem bark*, Musa sapientum* unriped fruits*, Bauhinia monandra* leaves*, Tetracarpidium conophorum* seeds*, Allium sativum* bulb, and *Gongronema latifolium* leaves were macerated in 1.5 L of distilled water for 24 hr according to extraction procedure practiced locally by users of these herbs. Dry powdered *Moringa oleifera* leaves (50 g) and powdered dry *Picralima nitida* seeds (70 g) were each macerated in 300 ml of distilled water. Each mixture was filtered, concentrated, and freeze‐dried. The freeze‐dried extracts were stored at −20°C until needed for in vitro analysis.

### 2.2 Chemicals

Acetic acid and HPLC‐grade acetonitrile were purchased from Merck (LiChrosolv GG, Darmstadt, Germany). Hydroxydiclofenac, desmethylomeprazole, 3‐hydroxyomeprazole, and hydroxycoumarin were obtained from Sigma‐Aldrich, St Louis, USA; 6‐hydroxytestosterone, dextrorphan, and desethylamodiaquine form BD Biosciences Discovery Labware, Bedford, USA; 5‐hydroxyomeprazole and omeprazole sulfone from Astra Zeneca, M¨olndal, Sweden; 1‐hydroxymidazolam from F. Hoffmann‐La Roche, Basel, Switzerland; phenacetin from ICN Biomedicals, Costa Mesa, USA. Hydroxybupropion was a free gift from Glaxo SmithKline Research Triangle, NC. Water used was purified by Simplicity 185 water purifier (Millipore, Molsheim, France). All other chemicals and reagents were of analytical grade.

### 2.3 CYP inhibition experiments

Human liver microsomes were obtained from a pool of liver samples from 25 male and female donors and contain 20 mg protein/ ml (Lot No: 99268 from BD Biosciences Labware, Bedford, MA). This was used for the metabolite profiling and CYP inhibition study. N‐in‐one approach (cocktail‐approach) for elucidating inhibition toward CYP‐specific model reactions was conducted with minor changes as described in earlier studies (Turpeinen, Jouko, Jorma, & Olavi, 2005; Tolonen, Petsalo, Turpeinen, Uusitalo, & Pelkonen, 2007; Showande, Fakeye, Ari, & Hokkanen, 2013). Briefly, the incubation mixture was made up of 0.3 mg microsomal protein/ml, 0.1 M phosphate buffer (pH 7.4), 1 mM NADPH, and nine probe substrates representing the major drug‐metabolizing CYPs. The specific probe substrate used for each CYP isoenzymes studied and the respective final concentration in the incubation mixture were as follows: acetaminophen (CYP1A2, 10 μM), coumarin (CYP2A6, 2 μM), bupropion (CYP2B6, 2 μM), repaglinide (CYP2C8, 5 μM), diclofenac (CYP2C9, 5 μM), omeprazole (CYP2C19 and CYP3A4, 5 μM), dextromethorphan (CYP2D6, 1 μM), midazolam (CYP3A4, 1 μM), and testosterone (CYP3A4, 5 μM). Each freeze‐dried aqueous herb extract was dissolved into methanol and added to the incubation mixture to obtain final concentrations of 0.001, 0.01, 0.1, 1, 10, 100, and 1000 μg/ml. The resulting reaction mixture (200 μl) contains 1% (v/v) methanol. This was preincubated in a shaking incubator block (Eppendorf Thermomixer 5436, Hamburg, Germany) for 6 min at 37°C prior to the initiation of the reaction with the addition of NADPH. Each reaction mixture containing different herb extracts or solvent control and other components was stopped after a period of 15 min by adding 200 μl of ice‐cold acetonitrile. Proteins were precipitated from the sample by cooling it in an ice bath. Supernatants collected from the samples were then stored at −20°C until analyzed. Prior to analysis, all incubation samples were thawed at room temperature, votex‐mixed, and centrifuged for 10 min at 10000 *g*. The experiments were performed in duplicate. The validated parameters for the methods were adequate for quantification ranges < 1%–300% of the forming metabolite concentrations with LOD 0.2–10 nM, accuracies 85%–115%, and precisions <15% at all concentrations, and with good autosampler stability (>95%) up to 48 hr. Positive controls (see Supporting Information data, Table S1), CYP isoenzymes inhibited, and concentration used in the N‐in‐one assay are as reported in the validated methods (Turpeinen et al., 2005; Tolonen et al., 2007; Showande et al., 2013). Spiked standard samples were not used for quantification of probe metabolites, but quantification based on relative peak areas was used (solvent control = 100%).

### 2.4 Liquid chromatography‐mass spectrometry conditions

The analysis of probe metabolites from CYP‐specific marker reactions was conducted with a LC/MS/MS method modified from earlier works (Turpeinen et al., 2005; Tolonen et al., 2007) and also reported by (Showande et al., 2013). Briefly, a Waters Acquity UPLC system (Waters Corp., Milford, MA) was used together with a Waters HSS C18 column (2.1 mm × 50 mm; 1.8 μm particle size) and an online filter at 35°C. The injection volume was 4 μl, and UPLC eluents were aqueous 0.1% acetic acid (pH 3.2, A) and acetonitrile (B). The gradient elution from 2%–65%–95% B was applied in 0–2.5–3.5 min, followed by column equilibration, giving a total time of 4.5 min/injection. The eluent flow rate was 0.5 ml/min. Data were acquired using a Thermo TSQ Endura triple quadrupole MS. Multiple reaction monitoring (MRM) mode using positive ion mode. For all compounds, the spray voltage was 4500 V, vaporizer temperature and transfer tube temperature were 400°C and 350°C, respectively. The CID argon pressure was set to 2.0 mTorr. The MRM transitions were as previously described (Tolonen et al., 2007; Turpeinen et al., 2005). For acetaminophen, hydroxyrepaglinide and 4‐hydroxydiclofenac MRM's were m/z 152 >  m/z 110, m/z 469 >  m/z 246, m/z 312 >  m/z 231, respectively. The instruments were controlled using Thermo Xcalibur 3.0.63 software.

### 2.5 Data analysis

#### 2.5.1 Determination of IC_50_

Fifty percent inhibitory concentration (IC_50_) values were determined graphically from the logarithmic plot of inhibitor concentration (concentrations of the aqueous extract of each herb) versus percentage of enzyme activity remaining after inhibition using GraphPad Prism 5.40 software (GraphPad Software Inc., San Diego, CA). The model equation (1) for the plot was.

(1) $$\%\;\text{Inhibition}=\frac{100}{\left(1+10^{\left(A-{\text{logIC}}_{50}\right)}\right)}$$

where A = log of concentration of aqueous extract of herb in the incubations.

#### 2.5.2 Classification of inhibitory potential of herb extract

The herb extracts were classified as potent, moderate, or weak inhibitor depending on the IC_50_ obtained (Gwaza et al., 2009; Sevior et al., 2010; Kong et al., 2011). Potent inhibitors have IC_50_ ≤ 10 μg/ml, moderate inhibitors have IC_50_ >10 μg/ml ‐ ≤100 μg/ml, and weak inhibitors have IC_50_ > 100 μg/ml. In this study, significant in vitro inhibition is considered as IC_50_ < 100 μg/ml (Sevior et al., 2010).

#### 2.5.3 Prediction of possibility of in vivo herb–drug interactions from in vitro data

Determination of molar IC_50_ values in herbal extracts is challenging thus representing the in vitro IC_50_ values in Liter/dose as described by Strandell, Neil, and Carlin (2004) gives insight into possibility of in vivo inhibition. The volume to which an estimated human unit dose of the aqueous extract of each herb should be diluted in vivo to give similar in vitro inhibitory concentration for each CYP isoenzymes was calculated using the equation (2)

(2) $${\text{IC}}_{50}\left(\frac{\text{Liter}}{\text{dose}}\right)=\frac{\text{Dose}(\text{mg})}{{\text{IC}}_{50}\left(\text{mg/L}\right)}$$

## 3 RESULTS

**Figure 1:**
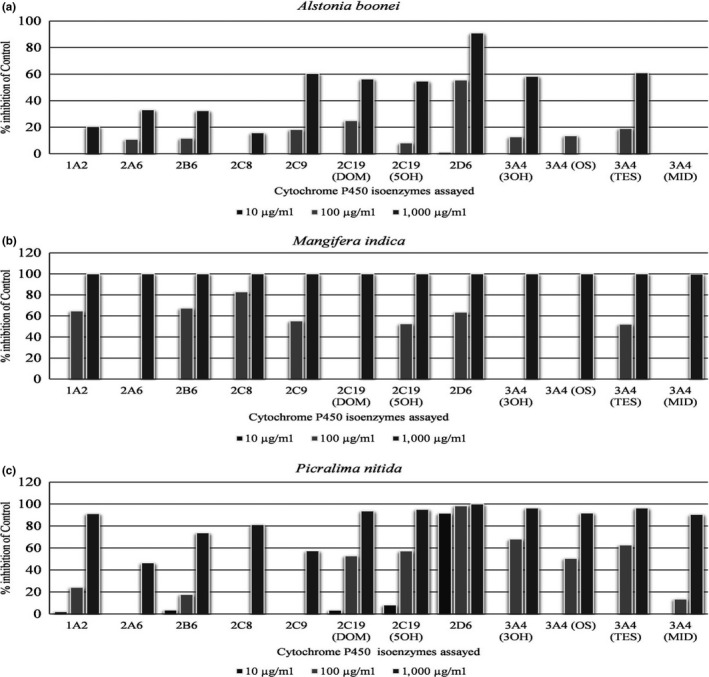
Percentage inhibition of the control by herbs with IC_50_ < 100 µg/ml–aqueous extracts of *Alstonia boonei* (a), *Mangifera indica* (b), and *Picralima nitida* (c). Each bar represents average of duplicate incubations performed at two separate experiments. DOM, desmethylomeprazole; 5OH, 5‐hydroxyomeprazole; 3OH, 3‐hydroxyomeprazole; OS, omeprazole sulphone; TES, 6β‐hydroxytestosterone; MID, 1‐hydroxymidazolam

Aqueous extracts of *Musa sapientum* unripe fruits*, Tetracarpidium conophorum* seeds, and *Allium sativum* bulbs did not produce significant inhibition of the CYP isoenzymes investigated at any of the concentrations used, IC_50_ > 1000 μg/ml. But *Gongronema latifolium, Bauhinia monandra,* and *Moringa oleifera* leave extracts showed weak inhibition of CYP1A2 with IC_50_ > 100 μg/ml (Table 2). *Alstonia boonei* stem bark aqueous extract also weakly inhibited CYP2C9, CYP2C19, and CYP3A4 but moderately inhibited CYP2D6 (Table 2). The inhibition of CYP2D6 was dose‐dependent inhibition (Figure 1a).

**Table 2:** Estimated in vitro IC_50_ values (μg/ml) for commonly used tropical medicinal herbs

CYP isoenzyme, metabolite monitored	*Alstonia boonei*	*Mangifera indica*	*Musa sapientum*	*Allium sativum*	*Tetracarpidium conophorum*	*Gongronema latifolium*	*Bauhinia monandra*	*Moringa oleifera*	*Picralima nitida*
CYP1A2, ACET	1104.95	**54.83**	1877.67	1431.57	1885.55	612.73	342.23	320.44	242.17
CYP2A6, 7‐OH‐COU	1916.01	222.72	ND	ND	4454.91	1103.52	1155.57	1343.99	862.60
CYP2B6, OH‐BUP	1213.23	**57.83**	1599.17	2318.16	3490.68	1096.56	1124.28	2280.15	469.00
CYP2C8, OH‐REPA	3209.33	**37.93**	27700.95	18745.49	ND	1255.15	389.37	1505.32	482.77
CYP2C9, OH‐DICL	507.96	107.48	48418.95	11285.64	ND	61940.47	1636.28	698.93	814.10
CYP2C19, DeM‐OME	356.20	115.48	4564.07	11897.19	12435.72	985.15	1329.80	1018.33	**84.90**
CYP2C19, 5‐OH‐OME	604.11	**88.03**	7129.64	7388.26	4683.85	700.66	1075.17	1108.75	**73.06**
CYP2D6, OdeM‐DEX	**77.19**	**67.39**	5783.82	12064.57	14054.21	1831.03	845.45	1929.26	**1.19**
CYP3A4, 3‐OH‐OME	460.99	104.56	8329.64	ND	5221.69	782.51	1441.96	3421.92	**45.58**
CYP3A4, SO2‐OME	973.88	125.79	17804.40	41358.77	8768.02	968.97	2291.01	7129.55	**74.68**
CYP3A4, 6β‐OH‐TES	529.63	**90.89**	10572.55	ND	5841.71	489.35	1030.02	2084.59	**57.39**
CYP3A4, 1‐OH‐MDZ	4411.11	203.63	ND	ND	82787.13	3384.92	10694.17	ND	222.64

IC_50_ value in bold represent significant inhibition of CYP isoenzymes with IC_50_ < 100 μg/ml, (ND) Not determined. Metabolites monitored the following: acetaminophen (ACET), 7‐hydroxycoumarin (7‐OH‐COU), hydroxybupropion (OH‐BUP), hydroxyrepaglinide (OH‐REPA), 4‐hydroxydiclofenac (OH‐DICL), 5‐hydroxyomeprazole (5‐OH‐OME), desmethylomeprazole (DeM‐OME), Dextrorphan (OdeM‐DEX), 3‐hydroxyomeprazole (3‐OH‐OME), omeprazole sulfone (SO2‐OME), 6β‐hydroxytestosterone (6β‐OH‐TES), 1‐hydroxymidazolam (1‐OH‐MDZ).

Aqueous extract of *Mangifera indica* stem bark showed weak inhibitory activities on CYP2A6, CYP2C19 and CYP3A4 but exhibited moderate inhibition of CYP1A2, CYP2B6, CYP2C8, CYP2C9, and CYP2D6 (Table 2). The plant extract displayed 100% inhibition of all the CYP isoenzymes investigated at a dose of 1000 μg/ml (Figure 1b).

*Picralima nitida* seeds aqueous extract demonstrated potent inhibitory activity on CYP2D6, with IC_50_ of 1.19 μg/ml, but moderately inhibited CYP2C19 and CYP3A4 (Table 2). These inhibitory activities were dose‐dependent (Figure 1c).

**Figure 2:**
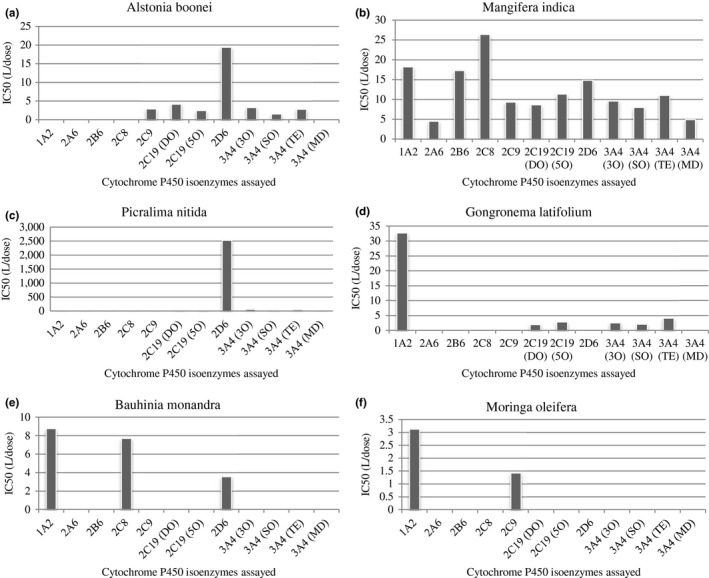
Inhibition of cytochrome P450 isoezymes by herbs with IC_50_ < 1,000 µg/ml–aqueous extracts of *Alstonia boonei* (a), *Mangifera indica* (b), *Picralima nitida* (c), *Gongronema latifolium* (d), *Bauhinia monandra* (e), and *Moringa oleifera* (f). Data represents IC_50_ in L/dose and each bar is an average of duplicate experiments performed on two different occasions. DOM–desmethylomeprazole, 5OH, 5‐hydroxyomeprazole; 3OH, 3‐hydroxyomeprazole; OS, omeprazole sulphone; TES, 6β‐hydroxytestosterone; MID, 1‐hydroxymidazolam

In order to estimate the possibility of in vivo inhibitory potential from the in vitro data, IC_50_ in L/dose was calculated according to the method described by Strandell et al. (2004). The IC_50_ values in L/dose are shown in Figure 2 for the herbs with IC_50_ < 100 μg/ml. *Picralima nitida* had an exceptionally high value of IC_50_ value of 2521.01 L/dose for CYP2D6 (Figure 2c). *Alstonia boonei* had IC_50_ of 19.43 L/dose for CYP2D6 (Figure 2a), *Mangifera indica* had IC_50_ > 14.00 L/dose for CYP1A2, CYP2B6, CYP2C8 and CYP2D6 (Figure 2b). Values of IC_50_ in L/dose unit for aqueous extracts of *Gongronema latifolium, Bauhinia monandra,* and *Moringa oleifera* aqueous extracts are shown in Figure 2d,e,f, respectively.

## 4 DISCUSSION

Drug–drug interaction or herb–drug interaction studies could be conducted as in vitro or in vivo studies, case reports, and human studies using human subjects. In vitro herb–drug interactions studies are mostly conducted for commonly used herbs to evaluate and predict potentially significant in vivo herb–drug interactions and to help design appropriate in vivo herb–drug interaction studies (Fasinu, Bouic, & Rosenkranz, 2012; Awortwe, Bouic, Masimirembwa, & Rosenkranz, 2013). According to Food and Drug Administration, 2017, in vitro drug–drug interaction (DDI) studies are designed to determine if a drug is a substrate, inhibitor, or inducer of metabolizing enzymes (Phase I or Phase II enzymes), transporter proteins (P‐gp, BCRP, OATP1B1, OATP1B3, OAT, OCT, and MATE) and also to evaluate whether the metabolite of a drug is a substrate or inhibitor of metabolizing enzymes or transporter proteins. These are referred to as metabolism‐based or transporter‐based drug interaction studies. Preclinical methods for predicting drug interactions use enzyme sources such as purified CYP450 isoenzymes, immortalized cell lines, recombinant P450 isoenzymes, liver slices, human microsomes, and hepatocyte cultures. Microsomes isolated from human hepatocytes have become the “gold standard” of in vitro experimentation for drug interactions and contain the CYPs in proportion to their in vivo representation. Pooled human liver microsomes were employed in this study to circumvent the large interindividual variability in CYP expression when microsomes from a single individual are used which may produce biased results. The N‐in‐one assay method employed in this study allows for rapid screening of many herbs or herbal products for inhibitory potential of major human CYP isoenzymes. This may help clinicians and patients to avoid concomitant use of herbs and drugs that may lead to potential or actual HDIs.

Three of the plants studied did not exhibit inhibitory potential on any of the eight major human CYP isoenzymes used even at the highest dose of the extract, that is, aqueous extracts of *Musa sapientum* unripe fruits*, Tetracarpidium conophorum* seeds, and *Allium sativum* bulbs. There are conflicting reports on the inhibitory potential of *Allium sativum* extracts on CYP isoenzymes. This herb was classified by Engdal and Nilsen (2009) as a noninhibitor of CYP3A4 using human c‐DNA baculovirus expressed CYP3A4. Using similar methods, Foster et al. (2001) reported in vitro inhibition of CYP 2C9, 2C19, 2D6, 3A4/5, and 3A7 by different types of *Allium sativum*. In another study, only two water‐soluble components of aged garlic were able to moderately inhibit CYP3A4 (Greenblatt et al., 2006). Also, aged garlic extract produced no significant inhibition of CYP isoenzymes in humans (Markowitz, 2003). Though we did not report any inhibition of CYP isoenzymes by *Allium sativum*, the discrepancies in these reports and ours may be due to the extraction procedure, assay method, concentration and type of the extract used and enzyme sources. In this study, aqueous extract of oven‐dried *Allium sativum* bulbs was used. There is no conclusive report of a significant in vitro inhibition of CYP isoforms by *Allium sativum* both from this study and others. Thus, the possibility of *Allium sativum* aqueous extract in vivo HDIs mediated by CYP isoenzymes may be remote as supported by our study and that of Markowitz (2003).

Aqueous extract of *Moringa oleifera* leaves showed weak inhibition of CYP1A2 and CYP2C9. This result is similar to the inhibition of CYP1A2 by ethanolic and aqueous extracts of *Moringa oleifera* leaves reported earlier (Taesotikul et al., 2010). Other studies reported inhibition of CYP3A4 (Ahmmed et al., 2015; Awortwe, 2015; Monera et al., 2008) and CYP2D6 by aqueous and/or ethanolic extract(s) of *M. oleifera* leaves (Ahmmed et al., 2015) which were not observed in this study. The possibility of in vivo inhibition of CYPs 1A2 and 2C9 by aqueous extract of *M. oleifera* is likely since the IC_50_ values in L/dose converted from μg/mL for CYP1A2 and CYP2C9 were 3.12 L/dose and 1.43 L/dose, respectively. These are higher than 0.88 L/dose estimated by Strandell et al. (2004) as the minimum required to elicit further investigation of in vivo inhibitory activity of the plant extract on CYP isoenzyme. However, in vivo study in human showed that *M. oleifera* did not affect the pharmacokinetic parameters of Nevirapine, a substrate of CYP2C9, CYP2D6, and CYP3A5 (Monera‐Penduka et al., 2017). Nonetheless, couse of *Moringa oleifera* leave extract with substrates of CYP1A2 and CYP2C9 should be further investigated for possibility of in vivo inhibition of these isoenzymes which may affect therapeutic outcome of coadministered medication.

Stem bark aqueous extract of *Mangifera indica* moderately inhibited CYP1A2, CYP2B6, CYP2C8, CYP2C9, and CYP2D6 (IC_50_ < 100 μg/ml) while others CYPs such as 2A6, 2C19, and 3A4 were weakly inhibited (IC_50_ > 100 μg/ml) by the extract. These results are similar to the inhibition of CYP1A2, CYP2C9, CYP2D6, and CYP3A4 by aqueous extract of *Mangifera indica* stem bark using human liver microsomes and primary hepatocytes reported by Rodeiro et al. (2008, 2013). *Mangifera indica* fruit and stem bark extract are commonly used by patients because of the folkloric claims and documented pharmacological activities in ameliorating the signs and symptoms of diabetes, treatment of malaria fever, menorrhagia, hypertension, and hypercholesterolemia (Ezuruike & Prieto, 2014; Awortwe, 2015; Gururaja et al., 2017). Most of the drugs used in the treatment and management of these diseases are metabolized by the major CYP isoenzymes, namely CYP1A2, CYP2C9, CYP2C19, CYP2D6, and CYP3A4 (Ogu & Maxa, 2000; Lynch & Price, 2007). Concomitant use of aqueous extract of *Mangifera indica* stem bark with substrates of these CYP isoenzymes especially CYP2C8, CYP2C9, and CYP3A4 may predispose the patient to clinically significant HDIs. Coadministration of *Mangifera indica* fruit with warfarin was found to increase the international normalize ratio (INR) significantly by 38% in thirteen male patients after 2–30 days of consumption (Monterrey‐Rodríguez, Feliú, & Rivera‐Miranda, 2002). This interaction was suggested to be due to the inhibitory effect of Vitamin A, contained in the fruit, on CYP2C19—an enzyme responsible for the metabolism (7‐hydroxylation) of the warfarin R‐isomer (Yamazaki, 1999). Aqueous extract of *Mangifera indica* bark also showed a weak inhibitory effect on CYP2C19 in the present study.

The potential for *Mangifera indica* stem bark aqueous extract to cause in vivo HDIs specifically with drugs with narrow therapeutic index is supported by the estimated IC_50_ values in L/dose which are higher than 5 L. As reported by Strandell et al. (2004), herb extracts with IC_50_ in L/dose greater than 0.88 L may cause in vivo inhibition of same CYP isoenzymes similar to the observed in vitro inhibitory potential of the herb on the same CYP isoenzymes.

Several studies have used Strandell et al. (2004) methods to predict possibilities of in vivo inhibition of CYP isoenzymes from in vitro data. For example, in vitro study by Sevior et al. (2010) predicted the possible absence of in vivo inhibition of CYP 2D6 and 3A4 by black cohosh and aqueous extract of valerian and inhibition of CYP2D6 by goldenseal. Earlier studies by Gurley et al. (2005, 2006, 2008) confirmed these predictions. Strandell et al. (2004) predicted the likelihood of potent in vivo inhibition of CYP3A4 by hypericum (St John's Wort) preparations; this also confirmed the report of Markowitz et al. (2000).

Currently, no study seems to be available on the in vitro or in vivo inhibitory activity of six of the herbs studied—aqueous extracts of *Picralima nitida* seeds*, Gongronema latifolium* leaves*, Tetracarpidium conophorum* seeds*, Musa sapientum* unripe fruits*, Bauhinia monandra* leaves*,* and *Alstonia boonei* stem bark. Our study may be the first report of the in vitro inhibitory potential of these herb extracts. *Gongronema latifolium* and *Alstonia boonei* may produce in vivo inhibitory activity on CYP1A2, CYP2C19 and CYP3A4 since the converted in vitro IC_50_ values are greater than 0.88 L/dose despite the weak in vitro inhibitory potential on these isoenzymes. Aside from its medicinal properties, *Gongronema latifolium* is a popular delicacy in African cuisine (Akinsanmi & Nwanna, 2015; Nwanna et al., 2016). This may increase the possibility of HDIs if drugs are taken after the consumption of a meal containing *Gongronema latifolium* vegetable. Aqueous extract of *Bauhinia monandra* leaves is likely to exhibit in vivo inhibition on CYP1A2, CYP2C8, and 3A4 with converted IC_50_ > 3.0 L/dose unit.

One of the problems of studying herb–drug interactions is the difficulty in identifying or quantifying the inhibiting components of the complex mixtures of phytochemicals in the herb. Because of this, it is difficult to quantify the molar IC_50_ value of these herbs; thus, the manufacturer recommended dose or the local or traditional dose is used to quantify the possibility of in vivo inhibition from in vitro IC_50_ values using the method described by Strandell et al. (2004). Though, it was concluded by Strandell et al. (2004) that herbs with converted IC_50_ > 0.88 L/dose unit should be investigated further for in vivo HDI. However, herbs with IC_50_ > 5 L/dose unit, the average human blood volume, are more likely to produce significant in vivo HDIs (Strandell et al., 2004).

*Picralima nitida* seem to be the most potent of the nine herbs studied with IC_50_ < 100 μg/ml for CYP2C19 and CYP3A4 and IC_50_ < 10 μg/ml for CYP2D6. The IC_50_ value converted into L/dose for CYP2C19, CYP3A4, and CYP2D6 far exceeded the 0.88 L/dose unit cutoff point by Strandell et al. (2004). With these values, this herb possesses the ability to elicit clinically significant in vivo HDIs, if used concomitantly with substrates of CYP2D6 such as amitriptyline, metoprolol, clozapine, flucainide, and retonavir (Lynch & Price, 2007). This isoenzyme is phenotypically expressed and the effect of HDIs may vary from one person to another. Thus, it is important to counsel patients not to coadminister this herb with any substrates of CYP2D6 until conclusive clinical data are available.

It should be borne in mind that the IC_50_ values are apparent values because of the complex mixture of herbs. The preparation of the herb extracts and the dose used also vary between localities. These may affect the computation of the IC_50_ values in L/dose unit and should be considered when interpreting these results. Also, despite the use of human liver microsomes in the in vitro study, extrapolation of the in vitro findings to in vivo may be limited by the in vivo bioavailability of the phytochemicals in the herbs. In spite of these result limitations, this study assisted in rapidly identifying herb extracts with potential for in vivo HDIs and those requiring further clinical studies.

## 5 CONCLUSION

Of the nine aqueous herb extracts evaluated in this study, *Picralima nitida, Alstonia boonei,* and *Mangifera indica* showed significant in vitro inhibition of several cytochrome P450 isoenzymes including CYP1A2, CYP2B6, CYP2C8, CYP2C9, CYP2C19, CYP2D6, and CYP3A4 with potential for significant clinical herb–drug interactions. The potent in vitro inhibitory effect of aqueous extract of *Picralima nitida* on CYP2D6 makes it a candidate for immediate further clinical investigations. However, before this is done, caution should be exercised by clinician and patients alike in recommending or coadministering aqueous extracts of *Picralima nitida* seeds with substrates of CYP2D6.

## DISCLOSURE

Authors have no conflict of interest to declare.

## ETHICAL STATEMENT

This study does not involve any human or animal testing.
