# Supplementary material for: Potential inhibition of major human cytochrome P450 isoenzymes by selected tropical medicinal herbs—Implication for herb–drug interactions
Source: Food Sci Nutr. 2018 Nov 19;7(1):44–55. doi: 10.1002/fsn3.789 (PMC6341161; doi:10.1002/fsn3.789)
Supplement: Supplementary file 1 [file FSN3-7-44-s001.docx]

**Supplemental data Table A: IC_50_ values of positive inhibitors**

| **Inhibitors** | **CYP isoenzymes** | **IC_50_ (µg/mL)** |
| --- | --- | --- |
| Fluvoxamine | CYP1A2 | 0.026 |
| Tranylcypromine | CYP2A6 | 0.365 |
| Ticlopidine | CYP2B6 | 0.026 |
| Quercetin | CYP2C8 | 17.47 |
| Quinidine | CYP2D6 | 0.011 |
| Sulphaphenazole | CYP2C9 | 0.063 |
| Fluconazole | CYP2C19* | 1.77 – 1.96 |
| Ketoconazole | CYP3A4* | 0.037 – 0.96 |

*Value varies depending on the probe substrate used
